# Microstructures and Properties of Ceramic Fibers of h-BN Containing Amorphous Si_3_N_4_

**DOI:** 10.3390/ma12233812

**Published:** 2019-11-20

**Authors:** Jing Tan, Min Ge, Shouquan Yu, Zhenxi Lu, Weigang Zhang

**Affiliations:** 1 State Key Laboratory of Multi-phase Complex Systems, Institute of Process Engineering, Chinese Academy of Sciences, Beijing 100190, China; tanjingewq@163.com (J.T.); gemin@ipe.ac.cn (M.G.); sqyu@ipe.ac.cn (S.Y.); zhxlu@ipe.ac.cn (Z.L.); 2 School of Chemical Engineering, University of Chinese Academy of Sciences, Beijing 100049, China

**Keywords:** polymer-derived ceramics, fibers, boron nitride, silicon nitride

## Abstract

Composite ceramic fibers comprising about 80 wt% boron nitride (h-BN) and 20 wt% Si_3_N_4_ were fabricated through melt-spinning, electron-beam curing, and pyrolysis up to 1600 °C in atmospheres of NH_3_ and N_2_, using a mixture of poly[tri(methylamino)borazine] (PBN) and polysilazane (PSZ). By analyzing the microstructure and composition of the pyrolyzed ceramic fibers, we found the formation of binary phases including crystalline h-BN and amorphous Si_3_N_4_. Further investigations confirmed that this heterogeneous microstructure can only be formed when the introduced ratio of Si_3_N_4_ is below 30% in mass. The mean modulus and tensile strength of the fabricated composite fibers were about 90 GPa and 1040 MPa, twice the average of the pure h-BN fiber. The dielectric constant and dielectric loss tangent of the composite fibers is 3.06 and 2.94 × 10^−3^.

## 1. Introduction

With the development of the aerospace industry, there is an urgent demand for high-performance wave-transparent materials such as antenna windows or domes equipped on hypersonic flight vehicles. These materials should have high thermal stability, good dielectric behavior, and excellent mechanical properties to meet the requirement of extreme working conditions. The fiber-reinforced ceramics matrix composites were widely used in these applications because of the machinability to complex shape and good mechanical reliability [1,2,3,4]. Reinforcing fibers in the composites play multiple roles, protecting the structure against heat and transmitting electromagnetic signals, that is to say, the performance of composites highly depends on the properties of reinforcing fibers.

Boron nitride (BN) ceramic fiber can be a good candidate for reinforcing fiber in wave-transparent applications due to its low density, high melting point, and low dielectric constant and loss tangent. In the past decades, BN ceramic fibers have been fabricated through the so-called polymer-derived-ceramic (PDC) process by many researchers [5,6,7,8,9]. In our previous study, we have prepared BN fibers with excellent dielectric properties by using poly[tri(methylamino)borazine] as a preceramic polymer [10]. However, the mechanical properties of the BN fiber were still weak which limited the wider application on structural materials. In order to improve the mechanical properties of BN fibers, Si_3_N_4_ is introduced to fabricate BN/Si_3_N_4_ composite fibers. Generally, Si_3_N_4_ fibers have better mechanical strengths than BN fibers, but the dielectric behavior of Si_3_N_4_ is inferior to BN [11,12]. Si_3_N_4_ ceramics can also be fabricated by the PDC route, after decarburization pyrolysis of polysilazane. Therefore, BN/Si_3_N_4_ composite fibers can be prepared by the blends of poly[tri(methylamino)borazine] and polysilazane. With the tailored composition of final BN and Si_3_N_4_, composite fibers are expected to combine the advantages of both kinds of ceramics and possess comprehensive performances with high mechanical properties and good dielectric behaviors.

In this paper, we have prepared BN/Si_3_N_4_ composite fiber by using blends of poly[tri(methylamino)borazine] and polysilazane, with other techniques the same as those of BN fiber. The microstructure and properties of the obtained fiber were analyzed.

## 2. Material and Methods

### 2.1. Preparation of Pre-Ceramic Polymer Blends

The pre-ceramic polymers of poly[tri(methylamino)borazine](PBN) and polysilazane(PSZ) were used for melt-spinning in this study. Their synthesis and properties have been reported [10,13], PSZ was synthesized by dichloromethylsilane and dichloromethylvinylsilane with a mole ratio of 1:1. The precursors of PBN and PSZ were first dissolved in toluene at 60 mass% and then mixed to form homogeneous hybrid precursor solutions with different proportions. The solutions were dried in a rotary evaporator at 60 °C for 2 h and then cooled to room temperature, producing the hybrid precursor solids. The final compositions of the derived ceramics were calculated according to the ceramic yield of each polymer. The ceramic yield of PBN and PSZ after decarburization pyrolysis is 40% and 63%, respectively.

### 2.2. Green Fiber Preparation

**Figure 1:**
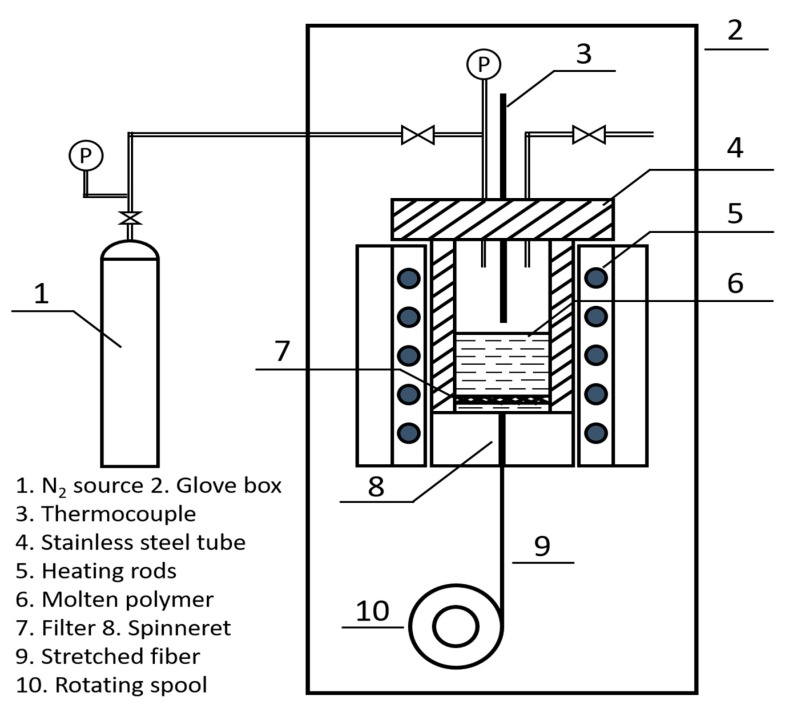
Schematic diagram of the melt-spinning apparatus.

Green fibers of pure PBN and hybrid PBN/PSZ with a mass ratio of 85:15 were prepared in a lab-scale melt-spinning apparatus (Figure 1) which is set up in a glove box filled with pure nitrogen. The polymer was charged into the spinning tube and then heated to the spinning temperature (about 140 °C) for 2 h. After the removing of the bubbles and an appropriate viscosity is obtained, the molten polymer was extruded through a spinneret with a diameter of 0.2 mm by increasing the pressure of N_2_. A filter was used to eliminate any unmelts. The extruded fiber was stretched and collected on a spool at a rotating speed of 8 m/s.

### 2.3. Fiber Curing and Pyrolysis

The obtained green fibers with spool were put on a rotating stainless steel cylinder and cured by the electron beam. The cylinder was set in a curing reactor filled with pure argon. The electron beam was generated with a linear accelerator. The following parameters were used in the current study: beam energy 2 MeV, beam current 0.5 mA for 30 min, 1 mA for 30 min, 1.5 mA for 30 min, and 2.5 mA for 60 min. A total dose of 6 MGy was deposited on the fibers. The cured fibers were pyrolyzed under tension in an alumina tube furnace. The furnace was heated to 1000 °C under flowing ammonia at a heating rate of 1 K/min, and then heated to 1600 °C under flowing pure nitrogen at 2 K/min. After holding at 1600 °C for 2 h, the furnace was cooled naturally to ambient temperature, producing white ceramic fibers.

### 2.4. Characterization

X-ray diffraction (XRD) patterns were measured using a PANalytical X’Pert-PRO diffractometer (Eindhoven, Netherlands) at 2*θ* = 10–90° with Cu Kα radiation (λ = 0.15406 nm at 40 kV and 30 mA). The d-spacings d_002_ and d_10_ of h-BN were calculated with Bragg’s law from the diffraction angle of the (002) peak and (10) peak, respectively. The average stack height L_c_ and size L_a_ were calculated from the (002) and (10) diffraction peaks with the Debye–Scherrer equations:

(1) $$\mathrm{Lc}=0.9\lambda /{\left(B^{2}-B^{\prime 2}\right)}^{1/2}\cos\theta$$

(2) $$\mathrm{La}=1.84\lambda /{\left(B^{2}-B^{\prime 2}\right)}^{1/2}\cos\theta ,$$

where *B* is the full width at half maximum (FWHM) intensity of the peak and *B’* is the contribution from the instrument.

The fibers were sprayed with an Au film and then observed with scanning electron microscopy (SEM) on an FEI Quanta 200 FEG system (JEOL, Tokyo, Japan). Transmission electron microscopy (TEM) samples were observed using an aberration-corrected ARM-200 microscope (JEOL, Tokyo, Japan) at an accelerating voltage of 200 kV. High-angle annular dark-field (HAADF) images were simulated using the xHREM program package, with inner and outer collection angles of 68 mrad and 150 mrad, respectively.

The element analysis of silicon and boron was analyzed by ICP-OES in a ThermoFisher iCAP6300 spectrometer (Waltham, MA, USA). Quantitative analysis of carbon, hydrogen, and nitrogen was determined by an Elementar Vario EL determinator (Langenselbold, Germany).

The dielectric properties of the fibers were determined at 9.5 GHz by an Agilent HP8722ES vector network analyzer (Santa Clara, CA, USA) at room temperature, according to the Chinese National Standard GB/T 5597–1999. Before the dielectric measurements, fibers were cut into short sections and molded into a cylinder sheet with a diameter of 60 mm and a height of 3 mm. These results were the average of five tests.

The tensile strength of the synthesized fibers was tested at room temperature using an Instron 5944 tensile tester (Norwood, MA, USA) equipped with a 10 N load cell, done at a constant cross-head speed of 0.05 mm/min. A series of 25 fibers with a gauge length of 25 mm was tested. The Weibull statistic [14] was used to estimate the average tensile strength of fibers for a failure probability of 0.632 and the average Young’s modulus was evaluated from these strain–stress curves.

## 3. Results and Discussion

### 3.1. Compositions and Morphologies of the Composite Fiber

The elemental compositions of the composite fiber during different steps are listed in Table 1. The green fibers show a weight loss of 55% and the carbon content decreased from 22.9 wt% to 0.2 wt% after the pyrolysis in an NH_3_ and N_2_ atmosphere. The obtained fibers have a nearly stoichiometric composition of (BN)(Si_3_N_4_)_0.05_.

**Figure 2:**
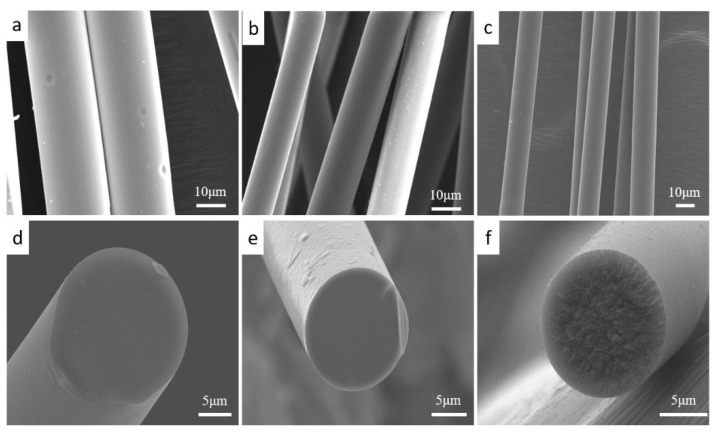
Scanning electron microscopy (SEM) images of the surfaces and cross-sections of the composite fiber: (**a**) and (**d**) show the green fiber after electron-beam curing, (**b**) and (**e**) show the fiber after decarburization pyrolysis at 1000 °C in NH_3_, and (**c**) and (**f**) show the fiber after heat treatment at 1600 °C in N_2_.

Figure 2a–f shows typical morphologies of the fabricated BN-Si_3_N_4_ composite fibers annealed at various temperatures. Figure 2a,d also show surface and cross-sectional morphologies of the green fibers after electron-beam curing, which show smooth surfaces and fracture sections without any apparent flaws such as voids or cracks. Before pyrolysis, the green fibers had an average diameter of about 22 μm. After pyrolysis at 1000 °C in NH_3_, the composite fibers retained their fibrous shape but shrank in diameter to about 14 μm (Figure 2b,e). The smooth and glass-like fracture section of the fibers may indicate that they remained in a glassy state. After heat treatment at 1600 °C in N_2_ (Figure 2c,f), the fiber core in the fracture cross-sections showed a coarse-grained texture (Figure 2f). However, the fiber shells still exhibited a glassy-like fracture, which should also correspond to low crystallinity.

### 3.2. Crystalline and Amorphous Phase Analysis

**Figure 3:**
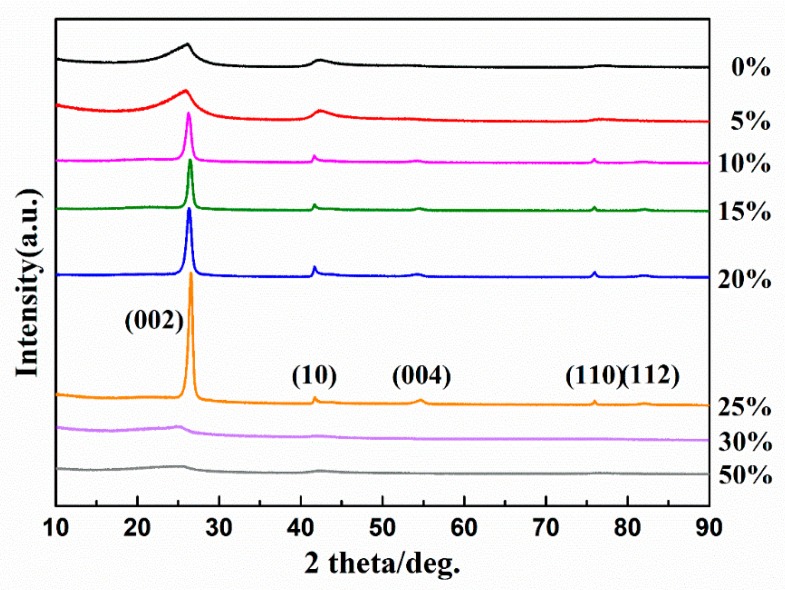
X-ray diffraction (XRD) patterns of BN-Si_3_N_4_ composite ceramics with various Si_3_N_4_ contents, all pyrolyzed up to 1000 °C in NH_3_ and then heated up to 1600 °C in N_2_ for 120 min.

To understand the further structure of the composite fibers, we analyzed the crystalline and amorphous phases by using XRD and high resolution transmission electron microscope (HR-TEM). Figure 3 shows the XRD patterns of the crystalline phases in the BN-Si_3_N_4_ composite ceramics with various contents of silicon-containing phases. Before these measurements, the samples were pyrolyzed at 1000 °C in NH_3_ and then heated to 1600 °C in N_2_ for 120 min.

In all samples, only h-BN appeared as crystalline, and no sample showed diffraction of silicon nitride: thus, Si_3_N_4_ existed in an amorphous phase even after annealing at 1600 °C in N_2_ for 120 min. Unsurprisingly, all polymeric pyrolysis routes to ceramic materials seem to pass through amorphous inorganic states, but these materials also transformed or decomposed into crystalline phases because of their thermodynamic instability [15,16]. Unexpectedly, the presence of an amorphous silicon-containing phase enhanced the crystallization of h-BN: the crystallinity of h-BN increased as the Si_3_N_4_ content increased up to 25 wt%. Also, the crystallinity of h-BN in the composite ceramics decreased dramatically when the Si_3_N_4_ content exceeded 30 wt%, and both h-BN and Si_3_N_4_ were fully amorphous at 50 wt%.

**Figure 4:**
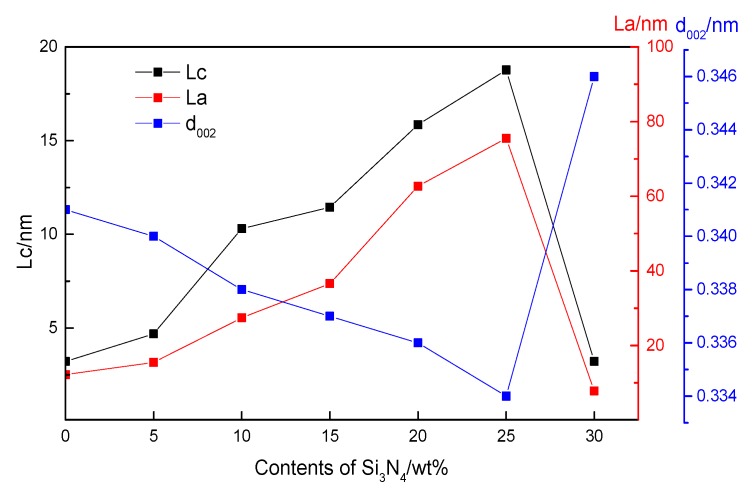
Crystalline parameters of h-BN with different Si_3_N_4_ contents.

Figure 4 shows the crystalline parameters of h-BN in various ceramics, determined from XRD and calculated with the Debye–Scherrer equation. The grain size of h-BN increased from 4 nm to 18 nm for L_c_, and from 3.6 nm to 75 nm for L_a_. Consistent with the grain size, the d-spacing was very high for pure h-BN (0.341 nm) and decreased nearly to its theoretical value (0.334 nm) when the content of silicon-containing phases increased to 25 wt%.

**Figure 5:**
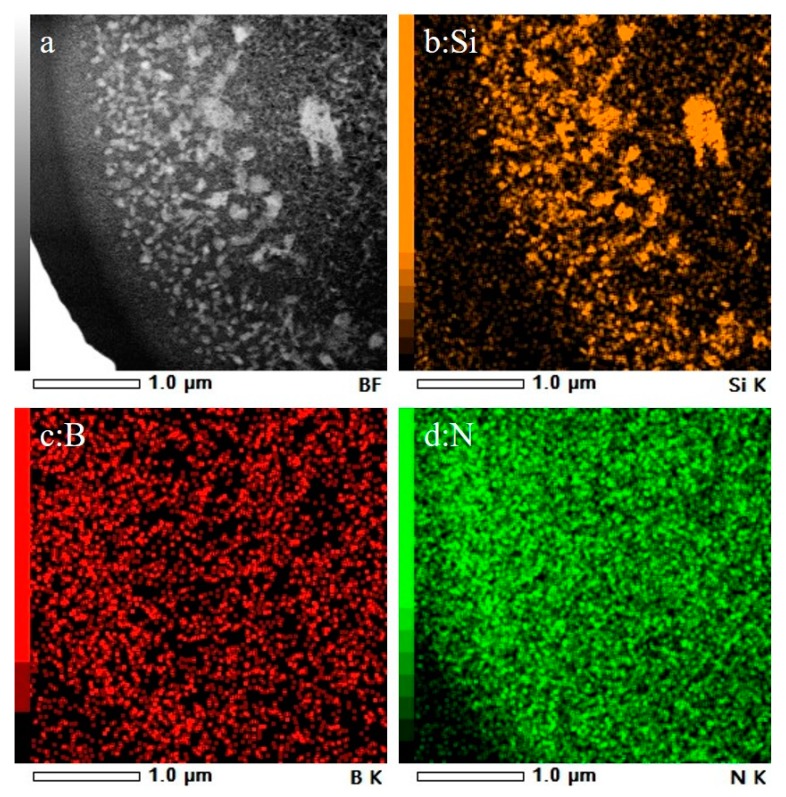
HAADF image (**a**) and element maps (**b**–**d**) in the composite fiber cross-section showing the constituent elements of Si (**b**), B (**c**), and N (**d**), revealing a heterogeneous distribution of Si and a homogeneous distribution of N.

Because the Si_3_N_4_ introduced in the composite ceramic fibers is amorphous even after annealing at 1600 °C, it cannot be distinguished by XRD or TEM. Figure 5a–d shows HAADF images based on the adsorption of chemical elements in the cross-section of the composite fiber. The bright spots representing Si (Figure 5a,b) all aggregated in the fiber core, while the dark fields representing B and N (Figure 5c,d) were homogeneously dispersed across the entire cross-section.

**Figure 6:**
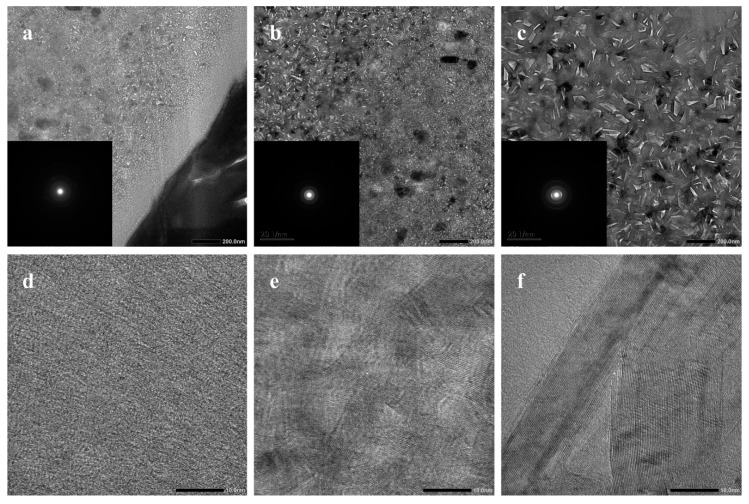
Cross-sectional HR-TEM images of the composite fiber taken at different locations: (**a**,**d**) show the shell, (**b**,**e**) show near the shell, and (**c**,**f**) show the core at low and high magnifications.

Figure 6 shows cross-sectional HR-TEM images of the composite fiber. The fiber shell comprised very small crystallites of turbostratic h-BN. However, near the shell, amorphous ceramics were crystallized and the grain size of h-BN grew to about 3–4 nm. Surprisingly, the fiber core showed very large crystals of h-BN (Figure 6c,f). The d_002_ calculated from the pattern was 0.334 nm, which agrees with the theoretical interplanar distance of crystalline h-BN. The average grain size was larger than 15 nm, which agrees well with the value calculated from XRD above. Large boron nitride crystals were mainly located in the core of the fiber where silicon-containing phases aggregated, which is in accordance with the XRD results.

### 3.3. Mechanical and Dielectric Properties

**Figure 7:**
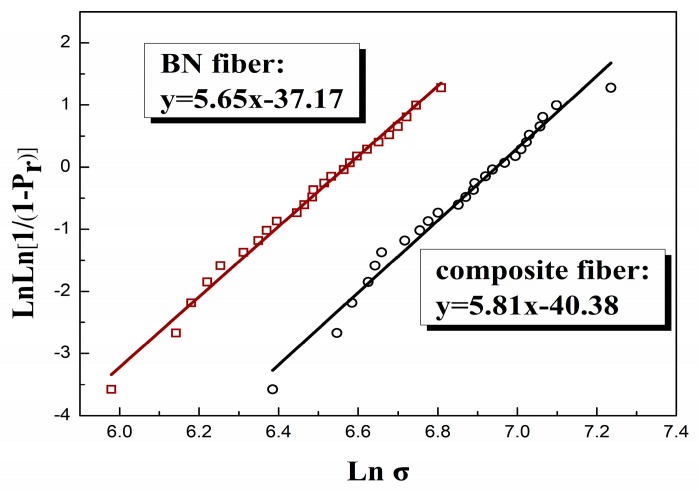
Weibull plot of failure strengths of these two fibers for a 25 mm gauge length: □ BN fiber, ○ composite fiber.

25 individual fibers were carefully chosen and submitted to mechanical tests at room temperature with a 25 mm gauge length. All the fibers showed brittle fracture. Figure 7 shows the Weibull plot of failure strength of two kinds of fibers for a 25 mm gauge length. The strength distribution of both fibers are nearly linear and the Weibull modulus of composite fibers (m = 5.81) is slightly higher than that of BN fibers (m = 5.65). The strength of BN fibers and composite ceramics fibers calculated for a failure probability Pr of 63% is 720MPa and 1040 MPa. The calculated average Young’s modulus of BN fibers and composite fibers were 42 GPa and 90 GPa, respectively. The mechanical properties of BN fibers and composite fibers were summarized in Table 2. Overall, the composite fibers appeared to have much higher failure strength and Young’s modulus with lower failure strain. The increase of fiber strength is owing to the extraordinary growth of h-BN crystals as well as the incorporation of high strength phase of Si_3_N_4_.

Table 3 summarizes the dielectric properties of BN fiber and composite fiber. The dielectric properties of other wave-transparent fibers [17] are also listed in Table 3. The dielectric constant and loss tangent of the BN/Si_3_N_4_ composite fiber were 3.06 and 2.94 × 10^−3^, slightly higher than those of the h-BN fiber. However, the dielectric behavior of the composite fiber is still satisfactory and better than that of quartz fiber and SiBN fiber. The excellent dielectric properties can be ascribed to the near stoichiometric composition of (BN)(Si_3_N_4_)_0.05_ with low carbon content. The combination of improved mechanical properties and excellent dielectric behavior indicates its promising potential for wave-transparent applications.

## 4. Conclusions

Composite ceramic fiber was fabricated by melt-spinning, electron-beam curing of a mixture of poly[tri(methylamino)borazine] and polysilazane and subsequent decarburization pyrolysis up to 1600 °C in atmospheres of NH_3_ and N_2_. The obtained fiber exhibited a heterogeneous microstructure comprising binary phases of crystalline h-BN and amorphous Si_3_N_4_. The extraordinary growth of h-BN crystals inside the ceramic fibers was found. This composite ceramic fiber showed better mechanical properties than the pure h-BN fiber and relatively good dielectric properties, which can be a promising candidate for reinforcement in wave-transparent applications. Further studies will focus on the mechanism of the growth of the h-BN crystals and the structure determination of the amorphous phase.

## Figures and Tables

**Table 1:** Elemental content of composite fibers during different steps.

Elemental Content (wt%)	Si	B	C	H	N
Green fibers	6.2	19.8	22.9	7.6	43.5
Fibers at 1000 °C	12.3	34.0	0.3	1.8	51.6
Fibers at 1600 °C	12.6	34.5	0.2	0	52.7

**Table 2:** Mechanical properties of two ceramic fibers.

Samples	Tensile Strength/MPa	Young’s Modulus/GPa	Failure Strain/%
BN fiber	720	42	1.71
Composite fiber	1040	90	1.15

**Table 3:** Dielectric parameters of the composite fiber and other fibers.

Samples	Dielectric Constant	tanδ
BN fiber	2.98	1.30 × 10^−3^
Composite fiber	3.06	2.94 × 10^−3^
Quartz fiber	3.12	3.60 × 10^−3^
SiBN fiber	4.36	4.20 × 10^−3^
